# New insights into immunomodulatory properties of lactic acid bacteria fermented herbal medicines

**DOI:** 10.3389/fmicb.2022.1073922

**Published:** 2022-11-28

**Authors:** Hongru Zhu, Lidong Guo, Dan Yu, Xiaowei Du

**Affiliations:** College of Pharmacy, Heilongjiang University of Chinese Medicine, Harbin, China

**Keywords:** lactic acid bacteria, herbal medicines, fermentation, immunomodulatory, bioactive substance, mechanism

## Abstract

The COVID-19 pandemic has brought more attention to the immune system, the body’s defense against infectious diseases. The immunomodulatory ability of traditional herbal medicine has been confirmed through clinical trial research, and has obvious advantages over prescription drugs due to its high number of potential targets and low toxicity. The active compounds of herbal drugs primarily include polysaccharides, saponins, flavonoids, and phenolics and can be modified to produce new active compounds after lactic acid bacteria (LAB) fermentation. LAB, primary source of probiotics, can produce additional immunomodulatory metabolites such as exopolysaccharides, short-chain fatty acids, and bacteriocins. Moreover, several compounds from herbal medicines can promote the growth and production of LAB-based immune active metabolites. Thus, LAB-mediated fermentation of herbal medicines has become a novel strategy for regulating human immune responses. The current review discusses the immunomodulatory properties and active compounds of LAB fermented herbal drugs, the interaction between LAB and herbal medicines, and changes in immunoregulatory components that occur during fermentation. This study also discusses the mechanisms by which LAB-fermented herbal medicines regulate the immune response, including activation of the innate or adaptive immune system and the maintenance of intestinal immune homeostasis.

## Introduction

The human immune system has gradually evolved to protect the body against foreign pathogens while effectively ensuring that it does not attack healthy tissue or endanger beneficial cohabitants. The immune system consists of an interactive network of lymphoid organs, cells, humoral factors, and cytokines and has three primary functions: immune surveillance, defense, and stabilization (Chaplin, 2010). When the immune system is functioning normally, it can reliably identify and remove invading viruses, senescent cells, and tumor cells. Over the past few years, studies have shown that disease occurrence and development are closely linked to immune function. Reduced immune activity is associated with severe infections, cancers and immunodeficiency diseases, while increased activity can lead to allergic, inflammatory and autoimmune diseases (Levy et al., 2017). COVID-19 pathogenesis typically includes an acute progressive pulmonary microcirculatory disturbance that is caused by the immune response to infection. Studies indicate that morbidity and mortality rates are higher among the elderly and immunocompromised (Forouzani-Haghighi et al., 2022). Thus, it is evident that the immune system plays a critical role in COVID-19 pathogenesis and prognosis.

Herbal medicines are regularly used due to their effectiveness in preventing and treating a wide range of medical conditions. While they have many active compounds, including polysaccharides, triterpenes, flavonoids, alkaloids, volatile oils, and phenols, the structure of particular active compounds makes it difficult for the human body to effectively absorb them and it is similarly challenging to purify them using conventional methods. Scientists have used a variety of physical, chemical and biotransformation methods to improve the content and bioavailability of the active substances in herbal drugs. In recent years, microbial conversion technology has been widely used to transform natural medicines due to its strong specificity, limited by-products, mild reaction conditions, and environmental-friendly processes. Fungi and bacteria are the most commonly biotransformed microbes. For example, *Fusarium* sp. C39, an endophytic fungus isolated from *Dioscorea nipponica*, can effectively transform *Dioscorea* saponins into diosgenin, a process thought to include glycolysis metabolism, a closed-loop reaction, dehydrogenation, and carbonylation (Huang et al., 2022). In addition, *Bacillus subtilis*-mediated fermentation of adlay (*Coix lacryma-jobi*) can significantly increase tetramethylpyrazine, γ-aminobutyric acid, triterpenes, phenols, flavonoids and, coixenolide levels (Wen et al., 2020). β-glucosidase-producing bacterial strains are often selected for biotransformation studies of rare saponins, isoflavones, and other substances (Geraldi et al., 2020; Lodha et al., 2021). Lactic acid bacteria (LAB) stand out among numerous alternative fermentation strains due to their safety and efficiency.

Lactic acid bacteria are generally recognized as safe (GRAS) microorganisms that are widely used in the food fermentation and biomedical industries. LAB fermentation can break down or convert undesired substrates into new active entities under the action of enzymes, thereby improving the bioactivity of natural medicines. For example, dried longan pulp fermentation using *Lactiplantibacillus plantarum* and *Leuconostoc mesenteroides* can boost its antioxidant activity by increasing the free and total phenolic content (Khan et al., 2018). In addition, fermentation mediated by *Lp. plantarum* ameliorates the therapeutic impact of *Danggui Buxue Tang* (DBT), a traditional herbal mixture, on type 2 diabetes mellitus by enhancing the inhibitory activity of α-glycosidase and suppressing the radical-scavenging and antiglycation of DPPH. Fermented DBT is shown to generate different flavonoid compounds than non-fermented DBT (Guo et al., 2020). Some natural compounds also influence the metabolic pathways of microorganisms, increasing the abundance of active secondary metabolites.

Recent studies have focused on the beneficial action of probiotic bacteria and their fermentation products on human health. LAB are shown to interact with human immune cells and regulate specific pathways involved in innate and adaptive immune responses to a variety of inflammatory diseases (Zhao et al., 2019). In particular, pre and probiotics in the fermentation system have a positive impact on the microorganisms and activity of the human gut, further supporting the immune system (Peters V. et al., 2019). For these reasons, LAB fermentation of herbal medicines is useful for enhancing the immunomodulatory activity of herbal products. The current study reviews the effects of LAB fermentation on the active substances in herbal drugs and the mechanisms by which these products activate the immune system.

## Immunoregulatory effect of lactic acid bacteria fermented herbal medicines

**Figure 1:**
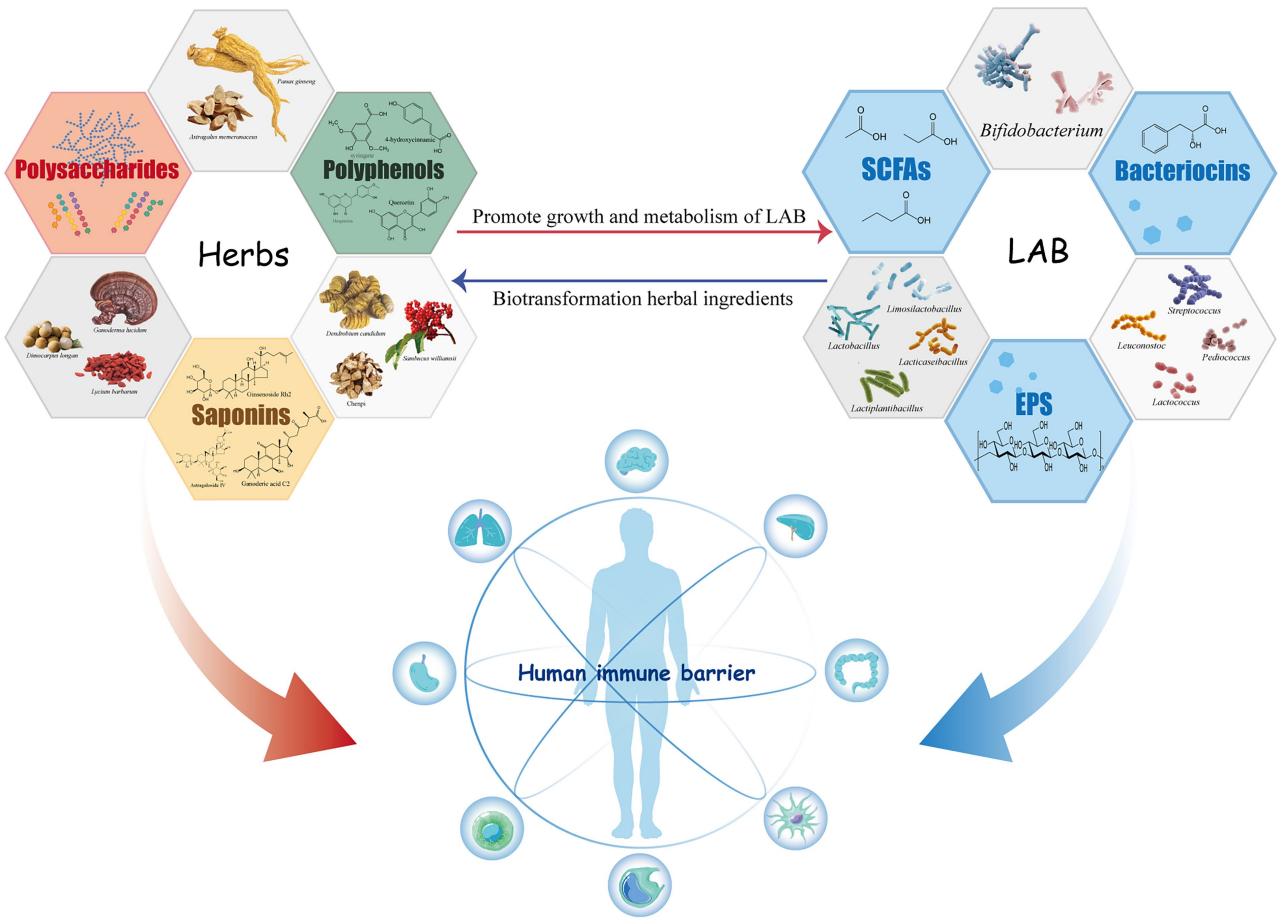
Immunomodulatory active components of lactic acid bacteria and herbal medicines and their interaction.

Lactic acid bacteria have a positive impact on the immunomodulatory activity of herbal medicine components during fermentation. The metabolic activity of LAB changes the content and structure of the active ingredients in herbal drugs, natural products similarly affect the growth status and metabolic process of LAB (Figure 1).

### Immunoregulatory effects of the active substances in herbal medicines

Herbal medicines have long been thought to contain a natural drug library that is effective against many human diseases including tumors, infections, and autoimmune diseases. Many ingredients in herbs are shown to benefit health, by activating the immune response. For example, the polysaccharides, saponins, and polyphenols found in herbs such as *Ganoderma lucidum*, *Lycium barbarum*, *Panax ginseng*, *Astragalus*, and *Dendrobium*, possess strong immunomodulatory activity.

#### Plant and fungal polysaccharides

Polysaccharides, such as the plant polysaccharide in *Astragalus membranaceus*, Longan, and *L. barbarum* (Yin et al., 2019), and the fungal polysaccharide in *G. lucidum* and *Poria cocos* (Yin et al., 2021), are common macromolecular immunomodulatory substances in herbal drugs. Several studies have shown that plant polysaccharides have multi-channel and multi-level regulatory effects on the immune system. They can activate several immune cell types, including T cells, B cells, macrophages, and natural killer cells, activate complement, and promote the production of cytokines, thereby regulating many components of the immune system. For example, plant polysaccharides can activate macrophages by recognizing and binding specific receptors on the cell surface, thereby initiating the immune response (Yin et al., 2019). Fungal β-glucans from *G. lucidum* or certain edible fungi can indirectly resist cancer cells or tumors by activating immune cells. In addition, β-glucan alleviates allergy by reducing the pro-inflammatory cytokines, IL-6 and TNF-α, and increasing the production of antioxidants (Murphy et al., 2020), a mechanism of action that is similar to that induced by pattern recognition receptors.

#### Saponins

Saponins are one of the secondary metabolite groups in medicinal herbs, exhibiting immunomodulatory properties using a variety of mechanism. Saponin compounds have been used to treat autoimmune diseases, allergic disorders and cancer. For example, Astragaloside IV can induce the differentiation of monocytes into M1 macrophages and initiate an anti-tumor immune response (Min et al., 2022). Glycyrrhetinic acid can alleviate allergic reactions in an asthma mouse model by inhibiting the production of the Th2 cytokines IL-5 and IL-13 (Kim et al., 2017). Ginsenoside Rh_2_ can enhance the infiltration and cytotoxicity of T lymphocytes in tumors, improving the cellular immune function of tumor-bearing mice (Wang et al., 2017). Ginsenoside compound K (CK) can inhibit the abnormal activation of T lymphocytes in mice with collagenous arthritis, and suppress memory T cells, preventing the immune system from rejecting heart transplantations (Wang et al., 2009; Liu et al., 2014).

#### Polyphenols

Polyphenols are plant-based organic compounds with a three-membered flavan ring structure. These substances are known for their health-promoting qualities, including scavenging free radicals, lowering oxidative stress, and reducing inflammation (Abbas et al., 2017). Phenolic compounds acting as antioxidants and anti-inflammatory agents help to mitigate oxidative stress, preserve the dynamic equilibrium of the intestinal inflammatory response and promote immunological tolerance (Williamson, 2017). Flavonoids and phenolic acids are the primary polyphenol family members. Flavonoids have a possible immunomodulatory effect on several illnesses, including autoimmune diseases and cancers (Ginwala et al., 2019). They have also been found to modulate key enzymatic functions of immune cells and mediate their immune-modulating activity (D'Arcy, 2022). Other natural phenolic compounds, such as resveratrol, also exhibit excellent immunostimulatory activities, increasing the infiltration of immune cells, regulating the phenotype, and promoting the secretion of related cytokines (Alesci et al., 2022).

#### Bioactive amino acids

Gamma-aminobutyric acid (GABA) is a kind of natural relaxant amino acid, that inhibits anxiety, depression, hypertension, and helps to regulate hormone secretion. GABA is found in the seeds, rhizomes, and tissue fluids of plants such as legumes, ginseng, and other herbal medicines. Dietary GABA improves non-specific immunity and reduces immune hyperactivity induced by lipopolysaccharide in adolescents (Zhang et al., 2022). Prior studies have shown that low physiological concentrations of GABA can inhibit T-cell growth by activating T-cell- specific GABA channels to suppress the immune response, a role that is promising in the treatment of autoimmune diseases (Bjurstöm et al., 2008; Prud'homme et al., 2015). Another study found that the oral administration of GABA can enhance the immune response under stress conditions (Abdou et al., 2006). Thus, appropriate supplementation with GABA may be beneficial to human health.

### Immunomodulatory effects and active substances of lactic acid bacteria

Lactic acid bacteria has several beneficial effects on human health, including its immunomodulatory activity. These functions have been confirmed in animal models and/or human clinical trials, and are shown to be closely related to the activity of LAB metabolites such as extracellular polysaccharides and short-chain fatty acids.

#### Exopolysaccharides

Probiotic bacteria secrete polysaccharides with a wide range of structures, compositions, and functions into the external environment. These are referred to as exopolysaccharides (EPS), most of which possess a broad spectrum of antitumor, immunomodulatory, glycemic control, and antioxidant effects (Nwodo et al., 2012). The immunoregulatory function of EPS strongly correlates with the chemical structure and configuration of these bacterial exopolysaccharides (Rajoka et al., 2020). More specifically, *Lactobacillus* EPS contain hydroxyl, phosphate, carbonyl, and other groups which promote their biological activities (Rajoka et al., 2020). In response to EPS from *Lacticaseibacillus rhamnosus* KL37, mouse peritoneal macrophages produce pro-inflammatory cytokines such as TNF-α, IL-6, and IL-12, and anti-inflammatory cytokines like IL-10, indicating that these compounds induce significant immunological activity (Ciszek-Lenda et al., 2011). Another study isolated and purified an acidic extracellular polysaccharide from *Lp. Plantarum* JLAU103, designated EPS103, with strong dual immunoregulatory activity (Wang et al., 2020). EPS103 was found to activate RAW264.7 macrophages while simultaneously reducing the excessive release and mRNA expression of several inflammatory factors, including IL-6, TNF-α, prostaglandin E2 (PGE2), NO, cyclooxygenase-2 (COX-2), and inducible NO synthase (iNOS) (Wang et al., 2020). These results suggest that LAB-specific EPS may act as immunomodulatory agents.

#### Short-chain fatty acids

Anaerobic bacteria in the colon ferment indigestible dietary fiber into short-chain fatty acids (SCFAs) such as acetate, propionate, and butyrate. These, in turn, serve as energy sources and participate in a variety of signaling processes (Russell et al., 2013). A recent study found that microbiota-derived SCFAs play a critical role in inducing IL-22 production from CD4^+^ T cells and innate lymphoid cells (ILCs) by inhibiting G-protein receptor 41 (GPR41) and histone deacetylase (HDAC) to maintain intestinal homeostasis (Yang et al., 2020). Another study found that both lactate and SCFAs could downregulate proinflammatory responses in intestinal epithelial cells and sentinel cells, including myeloid cells, by inhibiting TLR signaling in a dose-dependent manner, thereby restoring gastrointestinal homeostasis (Iraporda et al., 2015). The SCFA, pentanoate, was shown to reduce T-cell mediated immunopathology in the intestine and brains of mice by promoting IL-10 production and suppressing Th17 cells. Thus, pentanoate may have potential in the treatment of inflammatory and autoimmune diseases (Luu et al., 2019).

#### Bacteriocins

As they proliferate, LAB creates antimicrobial proteins or polypeptides called bacteriocins, which have been used as both food preservatives and treatments for foodborne infections (Zacharof and Lovitt, 2012). The use of bacteriocins as possible therapeutic antibiotics or immunomodulators is gaining greater attention (Huang et al., 2021). Bacteriocins produced by probiotics can also have a positive effect on the immune system. In addition to reducing the host inflammatory response by directly antagonizing pathogens, some bacteriocins can activate immune cells and enhance immunity (Peters V. et al., 2019). Nisin, produced by *Lactococcus lactis*, is shown to enhance macrophage-induced IL-12 production (Moein et al., 2018), modify the levels of inflammatory factors in a bidirectional manner, and maintain the immunological balance (Małaczewska et al., 2019). Sublancin, an S-linked glycopeptide, is also found to enhance macrophage function and promote CD4^+^ and CD8^+^ cell proliferation (Wang et al., 2018; Wang J. et al., 2019). This glycopeptide can inhibit nuclear factor-κB (NF-κB) activation to relieve intestinal inflammation (Hu et al., 2018). G protein-coupled hydroxy-carboxylic acid receptors (HCARs) regulate immune function and energy balance when metabolism and dietary conditions change (Offermanns, 2017). D-3-phenyllactic acid (D-PLA), an antibacterial metabolite produced by LAB, specifically recognizes the human and ape-specific HCA3 receptor and transmits signals to immune cells to induce immune responses (Peters A. et al., 2019). However, whether HCA3 activation by D-PLA can affect monocyte phagocytosis or differentiation into macrophages requires further study.

### Interaction between lactic acid bacteria and herbal medicines: Promoting effect of immunomodulation

LAB and herbal ingredients interact bidirectionally during fermentation. This process improves the biological activities of natural products by inducing the synthesis of various immunoreactive compounds in herbs or modifying natural molecules to enhance their immunoregulatory effects (Table 1). At the same time, some components in herbs can promote the growth and production of beneficial metabolites from LAB. The relationship between LAB fermentation and herbal medicines and their impact on the host immune system is shown in Figure 1.

**Table 1:** The immunomodulatory effect of herbal medicines fermented by lactic acid bacteria.

Herbal medicines	Strains	Active ingredients change	Models	Immunomodulatory effects	Molecular mechanism	References
Red Ginseng	*Lacticaseibacillus paracasei* subsp. *paracasei*	Ginsenoside Rb_1_ → F_2_ and CK	RAW 264.7 cells and mice	Macrophages (NO and TNF-α↑), splenocytes (TNF-α, IL-6↑)	-	Park et al. (2014)
	*Lactiplantibacillus plantarum* M-2	Total sugar ↓ (85.5 → 44.1 mg·mL^−1^), uronic acid ↑ (534.3 μg·mL^−1^), ginsenoside metabolites: Rh_1_, Rh_2_, Rg_5_, Rk_1_, Rg_2_ and Rg_3_ ↑ (4637.0 → 7581.1 μg·mL^−1^)	Mice and human	Macrophage phagocytosis↑, TNF-α ↑, IgM and IgG ↑	–	Kim et al. (2011)
	*Bifidobacterium animalis* subsp. *lactis* LT 19–2	Ginsenoside Rb_1_, Re, Rc, and Rb_3_ ↓, Rd., Rh_1_, F_2_ and Rg_3_ ↑	RAW 264.7 cells, mice splenocytes and BMDM	Macrophages (TNF-α and IL-6 ↑), splenocyte proliferation ↑, BMDM (TNF-α ↑)	Induce phosphorylations of ERK, P38, JNK and NF-κB	Kim et al. (2019)
	*Lacticaseibacillus paracasei* HY7017	Ginsenoside Rb_1_ → Rg_3_	RAW 264.7 cells, splenocytes, NK cells and mice	Macrophages (NO, TNF-α and IL-6↑), splenocytes (IL-12 and IFN-γ↑), restore WBC counts, IL-2 and IFN-γ ↑, NK cells↑	–	Mo et al. (2021)
Flower-buds of *Panax ginseng*	*Lactiplantibacillus plantarum* and *Lacticaseibacillus casei*	Total polyphenols↑ (27.41 → 44.22 mg·g^−1^), ginsenoside Rb_1_, Rg_2_, Rg_3_, Rh_1_ and Rh_2_↑ (64.47 → 93.70 mg·g^−1^)	T lymphocytes	IL-1β ↓, IL-2 and TNFSF14 mRNA ↑	–	Kim et al. (2015)
Ginseng	*Lactiplantibacillus plantarum* KP-4		RAW 264.7 cells	ALT and AST ↓, IL-6, TNF-α and IL-1β ↓, claudin-1 ↑	Inhibit TLR4/MAPK signaling pathway	Fan et al. (2021)
Hydroponic Ginseng	*Levilactobacillus brevis* B7		RAW 264.7 cells	TNF-α and iNOS mRNA ↑	–	Song et al. (2021)
Longan	*Lactiplantibacillus plantarum* GIM 1.191	Neutral sugar and uronic acid ↓, Mr. (221.63 → 109.62 kDa), glucose↓ (38.71%), galactose ↑ (11.28%) and arabinose ↑ (11.80%)	RAW 264.7 cells	NO and IL-6 ↑	–	Huang et al. (2019)
Gynostemma	*Lactiplantibacillus plantarum*	Gypenoside A and XLIX, Ginsenoside CK and F_2_ ↓, Rg_3_↑	Mice	Growth performance and immune organ index ↑, IgA, IgG and IgM ↑, IFN-γ, IL-2, IL-4 and IL-6 ↑	-	Du (2021)
Coix Seed	*Lactiplantibacillus plantarum NCU137*	β-glucan, cordycepin and SCFAs ↑	Mice	Spleen index ↑, CD4^+^/ CD8^+^ ↑, IgM and IgG ↑, TNF-α and IL-6 ↓	-	Wang et al. (2022)
*Cordyceps Militaris*	*Pediococcus pentosaceus* GRC-ON89A	Total polysaccharides ↑ (11.65%), protein ↓ (8.9%), Glc: Gal: Ara = 27.92: 5.20: 2.86 → 23.45: 2.50: 3.16, Mw↓	RAW 264.7 cells and mice	NO ↑, macrophage phagocytosis↑, TNF-α and IL-10 mRNA ↑	Upregulate TLR-2 and FcγR-mediated signaling	Kwon et al. (2018)
Astragalus	*Streptococcus alactolyticus* FGM		Mice	SIgA α-chain and pIgR gene ↑, sIgA ↑, IgA, IgG and IgM ↑, IL-2 and IFN-γ ↑, T-AOC and SOD ↑, TJ/AJ protein ↑	-	Liang (2019)
			Mice	Organ index ↑, TNF-α and IL-1β↓	–	Zong et al. (2018)
		Yield of polysaccharides ↑ (1.91 → 3.31%), purity of polysaccharides ↑ (88.5 → 91.3%)	BMDC	Promote the maturation of BMDC, IL-12, TNF-α, IL-1β and IL-6 ↑ and T cells↑	Activate MAPK-JNK, P38 MAPK and NF-κB pathways	Bian (2017)
*Ganoderma lucidum* fruiting body	*Lactobacillus acidophilus* and *Bifidobacterium breve*	Ganoderic acid A (GA) → GC2, total polysaccharides ↑	Mice	Regulate gut microbes, IL-17 and IFN-γ ↓, TNF-α, occludin, ZO-1, CD4^+^ and CD8^+^T cells, CD4^+^/CD8^+^ ↑	–	Li et al. (2021)
*Dendrobium candidum*	*Lactiplantibacillus plantarum* SHYZ001	P-hydroxycinnamic acid, p-hydroxybenzoic acid and syringic acid ↑	*Danio rerio*	Regulate gut microbes, SCFAs, MUC2, ZO-1, occluding, SOD, GSH-Px, IL-4 and IL-10 ↑	–	Gong et al. (2020a)
*Lycium Barbarum*	*Lactobacillus helveticus*	Polysaccharide ↑ (29%) and polyphenol ↓ (90.96%)	Mice	Spleen lymphocytes proliferation ↑, immune organ index ↑, IL-13, IL-8, IL-12, IL-17 and IFN-γ ↑, TLR-4, MUC-1, MUC-2 ↑	–	Li (2021)

BMDC: bone marrow-derived dendritic cells, WBC: white blood cell, TNFSF: tumor necrosis factor superfamily, ALT: alanine aminotransferase, AST: aspartate aminotransferase, T-AOC: total antioxidant capacity, SOD: superoxide dismutase, ZO-1: zonula occludens-1, MUC: mucoprotein, GSH-Px: glutathione peroxidase, ERK: extracellular regulated protein kinases, JNK: c-Jun N-terminal kinase.

#### Effect of lactic acid bacteria fermentation on herbal medicines

##### Effect of fermentation on plant and fungal polysaccharides

Fermentation is an effective strategy for increasing polysaccharide content in plants or fungi, inducing the structural breakdown of plant or fungal cell walls, and promoting the liberation or synthesis of immunoreactive ingredients. Fungi cell walls are primarily composed of high molecular weight chitin, which is difficult for the human digestive system to break down. Chitinase produced by microbial fermentation helps to disrupt the fungal cell wall matrix and facilitate polysaccharide extraction (Brurberg et al., 1994). *Lactiplantibacillus* fermentation effectively promotes the release of immune active substances such as *G. lucidum* polysaccharides by destroying the cell wall of spores, thereby improving the ability of these compounds to regulate immunity. More specifically, *Lp. Plantarum*-induced fermentation of *G. lucidum* spores for 48 h effectively destroys the spore wall structure, at a rate of 52.38% (Nguyen and Nguyen, 2015), without altering the stability of the active polysaccharide structures (Chaiyasut et al., 2010).

The immunoregulatory activity of polysaccharides depends to a great extent on their structural characteristics such as molecular weight, monosaccharide composition, α/β-configuration, conformation and glycosidic bond (Chen and Huang, 2018). Polysaccharides can activate intracellular signaling pathways, promote gene expression of immune molecules such as cytokines, and avtivate the immune system by recognizing the corresponding pattern recognition receptors (PRRs), such as dectin-1, complement receptor 3 (CR3) and Toll-like receptors (TLRs) on the surface of immune cells (Jiang et al., 2010; Yin et al., 2019). Glycosidase secreted by LAB can degrade the molecular chain of polysaccharides, which involves the decomposition of insoluble macromolecular chains into small oligosaccharide fragments with different forms. This may allow polysaccharides to bind to and activate immune cell receptor proteins. Polysaccharides fermented by LAB may also produce some functional oligosaccharides such as chitosan, algal, mannan, and galacto-oligosaccharides, which are beneficial to human intestinal health (Patel and Goyal, 2011) by modulating gut flora, promoting the production of short-chain fatty acids, strengthening the intestinal mucosal barrier, alleviating inflammation, and improving intestinal immunity (Cheng et al., 2019; Ma et al., 2020; Carvalho et al., 2021; Zhang N. et al., 2021).

*Streptococcus alactolyticus*-mediated fermentation of *Radix astragali* (RA), a traditional herb derived from the root of *Astragalus membranaceus*, significantly increases the total sugar contents of RA (Liang, 2019). Further analyses using HPLC, HPGPC and FT-IR showed that the average molecular weight of fermented *Astragalus* polysaccharides (FAPS) was lower than a non-fermented control, and FAPS with the structure of (1 → 4)-α-D-glucan appear to have strong immunopotentiation (Li et al., 2020). An antioxidant activity assay found that the scavenging rate of FAPS DPPH free radicals (FR) (26.40%) was higher than of the rate of APS (20.81%), and the former showed an enhanced ability to restore intestinal epithelial cell wall barrier function and resolve intestinal mucosal damage caused by cyclophosphamide (Liang, 2019). Another study reported that *Limosilactobacillus fermentum*-mediated fermentation effectively modified some of the physicochemical and biological properties of longan pulp polysaccharides (LP) (Huang et al., 2019). Fermented longan polysaccharide (LP-F), which has a 50% lower molecular weight than LP, was found to contain less neutral sugar, uronic acid, and glucose, but more arabinose, galactose, rhamnose, and mannose. LP-F also had higher solubility, lower apparent viscosity, and smaller particle size, meanwhile exhibited stronger immunomodulatory and prebiotic activities, with increased induction of nitric oxide (NO) and interleukin (IL)-6 by macrophages and higher production of intestinal probiotics (Huang et al., 2019). Thus, the fermentation modification of plant or fungal polysaccharides by LAB is an important way to improve its immunomodulatory activity.

##### Effect of fermentation on saponin compounds

Microbiota in the human intestine is shown to deglycosylate large molecular weight saponins with long sugar chains into small molecular weight saponins with <2, that exhibit stronger pharmacological activity. Recent studies have focused on the role of LAB in saponin biotransformation. By producing β-glucosidase, LAB can convert precursor saponins into rare secondary saponins through biochemical reactions such as deglycosylation, oxidation and dehydration, thereby improving their biological activity.

During *Lp. plantarum* M2-mediated fermentation, levels of the ginsenosides, CK, Rh_1_, Rh_2_, Rg_5_, Rk_1_, Rg_2_ and Rg_3_, in red ginseng (RG), which are easily absorbed bythe small intestine, increase by 67.5% (Kim et al., 2011). Fermented red ginseng (FRG) is also found to exert a much stronger anti-tumor response *in vivo* by enhancing the mucosal immune system. Indeed, the FRG inhibition rate at a dose of 500 μg·mouse^−1^ was 21% higher in the fermented than in the non-fermented group (Kim et al., 2011). Immunoglobulin A (IgA) in the serum of healthy subjects was also higher (5.14 mg·mL^−1^) after FRG administration than at baseline, while unfermented RG had the opposite effect (−14.50 mg·mL^−1^). These findings indicate that FRG can effectively regulate the humoral immune response (Kim et al., 2011). Park et al. (2014) found that Woongjin fermented red ginseng, which is produced by *Lacticaseibacillus paracasei* subsp. *paracasei* fermentation, has a stronger immunostimulatory effect than non-fermented RG, by increasing immune-related cytokine production and stimulating splenocyte proliferation. Further HPLC analysis found that RG extract significantly increased the content of ginsenoside metabolites, F_2_ and CK, after fermentation. CK, which does not occur naturally in ginseng, is a potent novel metabolite produced from microbial fermentation and transformation (Park et al., 2014). Kim et al. (2019) also reported that the levels of several functional aglycones, including Rd., Rh_1_, F_2_, and Rg_3_, in RG were increased by fermentation with *Bifidobacterium animalis* subsp. *lactis* LT 19–2. FRG had a stronger impact on macrophage activity than RG, demonstrated by higher tumor necrosis factor (TNF)-α and IL-6 production. A recent study found that multi-strain fermentation with *Lactobacillus acidophilus* and *Bifidobacterium breve* transformed *G. lucidum* triterpenoids, including lucidenic acid A, lucidenic acid N, ganoderic acid J, and ganoderic acid AM1 into organic acids, such as ganoderic acid C2 (Li et al., 2021). The fermentation product had a greater impact on the immune function of themucosal barrier than the non-fermented extract and reversed gut microbiota dysbiosis in immunosuppressed mice (Li et al., 2021). Thus, modifying the structural makeup of saponins through LAB fermentation is an effective strategy for boosting the immunomodulatory effects of natural medicines.

##### Effect of fermentation on polyphenol compounds

Fermentation is an effective way to increase the polyphenol content of plants. In addition to promoting the structural collapse of plant cell walls using microbial enzymes, fermentation may modify the metabolic pathways of microorganisms, resulting in the liberation or synthesis of novel bioactive substances (Hussain et al., 2016). Gong et al. (2020a) reported that *Lp. plantarum* SHYZ001-mediated fermentation can change the phenolic contents of *Dendrobium candidum*. HPLC-based analyses showed that the levels of the phenolic acids, 4-hydroxycinnamic acid and syringat, in fermentation broth were increased by 42.2914 μg·g^−1^ and 12.3510 μg·g^−1^, respectively. Non-targeted metabolomics analysis also found that the phenylpropanoid metabolic pathway, the primary pathways for the formation of phenolic compounds, is up-regulated during fermentation (Gong et al. 2020a). In addition, some LAB strains, including *Levilactobacillus brevis*, *Limosilactobacillus fermentum* and *Lp. plantarum*can metabolize phenolic acids through divers enzymes, such as polyphenol oxidase, decarboxylase and reductase (Filannino et al., 2015). After fermentation with *Lacticaseibacillus rhamnosus* and *Lp. plantarum* strains, the content of polyphenolic compounds such as quercetin-3-O-rutinoside, hydroxycinnamic acids, flavonols and anthocyanins was increased and several phenolic metabolites, including dihydrocaffeic acid and catechol, in elderberry juice are produced (Ricci et al., 2019).

Flavonoids in herbal medicines can undergo structural alteration during fermentation, that increases their immunoregulatory activity. In nature, they are frequently found in glycosylated forms, such as baicalin, apigenin, luteolin, and icariin. Native flavonoid glycosides cannot be directly absorbed by the human organism and must be first hydrolyzed into aglycones by intestinal enzymes or colonic microflora (Manach et al., 2004). Thus, flavonoid bioactivity is highly dependent on bacterial metabolism. During fermentation, β-glucosidase and other hydrolases produced by LAB may contribute to the cleavage of inter-sugar bonds, releasing matching glycosides that are hydrolyzed to release phenolic aglycon moieties (Martins et al., 2011). *Lactiplantibacillus pentosus* NGI01, a LAB strain isolated from kimchi, was found to convert 69.1 and 19.4% of the flavonoid glycosides, hesperidin and rutin, respectively, into their aglycone forms, hesperetin and quercetin, under optimal biotransformation conditions (Park et al., 2021). Another study showed that transformed strains of *Limosilactobacillus mucosae* INIA P508 were capable of effectively catalyze the deglycosylation of lignans, isoflavonoids, flavones, and flavanones by heterologous production of the β-glucosidase GLU913 (Gaya et al., 2020). Esterase enzymes are also widespread in *Lp. plantarum* strains, promoting flavonol glycoside metabolism in plant matrices (Esteban-Torres et al., 2013). Fermentation of cactus pear pulp using *Lp. plantarum* strains and *Levilactobacillus brevis* POM4 produced two flavonoid derivatives, kaemferol and isorhamnetin, which enhanced their antioxidant and immune-modulating properties (Filannino et al., 2016). These findings indicate that the use of LAB with enough hydrolase activity to modify flavonoid structure and improve its bioavailability is one way to improve the immunomodulatory activity of natural products.

##### Effect of fermentation on bioactive amino acids

Bioconversion techniques that utilize microorganisms have attracted increased attention as a mechanism to produce GABA. Glutamic acid, a GABA precursor, is widely found in natural plants and animal drugs. LAB-derived glutamic acid decarboxylase and coenzyme factor, 5′-pyridoxal phosphate, can convert glutamic acid into GABA. Kim et al. (2014) reported that *Gastrodia elata* Bl. fermentation induced by the co-cultivation of *Levilactobacillus brevis* GABA 100 and *Bifidobacterium bifidum* BGN4, large quantities of organic acids and GABA were generated, and 75.3% of the glutamate was converted into GABA. *Portulaca oleracea* L. fermentation using *Levilactobacillus brevis* POM4 also led to high amounts of GABA production (*ca.* 400%) and 30% lower levels of oxalic acid, an anti-nutritive factor (Filannino et al., 2017). Yoo et al. (2022) also reported that fermentation of deer antler velvet with *Lacticaseibacillus rhamnosus* LFR20-004 and *Latilactobacillus sakei* LFR20-007 augmented the concentration of bioactive amino acids, such as sialic acid and GABA. Fermented antlers exerted potent immune-enhancing activity by stimulating in the production of IL-6, IL-10, IFN-γ and TNF-α, possibly due to an increase in these bioactive molecules (Yoo et al., 2022).

#### Effect of herbal medicines on lactic acid bacteria

Different natural products are thought to affect LAB growth in distinct ways. In general, polysaccharides can be used as a source of energy to facilitate bacterial growth, reproduction and metabolic processes, including the production of SCFAs. SCFAs are produced by glycolysis through specific enzymes from a range of bacteria (Macfarlane and Macfarlane, 2003). Their production is associated with multiple factors, including strain species, growth status, and substrate properties (Özcelik et al., 2016; Annunziata et al., 2020; Hadinia et al., 2022). Fermentation *in vitro* is recognized as a feasible strategy for obtaining large amounts of SCFAs. The SCFAs, acetic, propionic, and butyric acid were produced by four LAB strains isolated from Korean individuals and fermented foods, of which *Bifidobacterium bifidum* MG731 induced 4,998.6 μg·g^−1^ SCFA production, *Bifidobacterium lactis* MG741 induced 2,613.9 μg·g^−1^, *Ligilactobacillus salivarius* MG242 induced 1,456.1 μg·g^−1^, and *Lp. plantarum* MG989 induced 630.2 μg·g^−1^ (Kang et al., 2010). In addition, fructan and galactan from *Polygonatum cyrtonema* were shown to be selectively utilized by *Bifidobacterium* and *Ligilactobacillus* probiotic strains, highlighting their growth-promoting properties (Zhang J. et al., 2021). *Polygonatum sibiricum* polysaccharides primarily composed of rhamnose, arabinose, and xylose, may be degraded into monosaccharides and produce acetic acid, propionic acid, and lactic acid by intestinal microbial fermentation (Luo et al., 2022). Sarep, a valuable ingredient obtained from orchid tubers, was shown to exhibit prebiotic potential because of its glucomannan content, providing a carbon source for the growth of *Bifidobacterium infantis* and promoting the production of its SCFA metabolites (from 1.27 g·L^−1^ to 1.94 g·L^−1^) (Usta-Gorgun and Yilmaz-Ersan, 2020). *G. lucidum* polysaccharides significantly increased the relative abundance of *Ligilactobacillus*, *Bifidobacterium*, and SCFAs in the intestine of colitis mice, by activating G-protein-coupled receptor 43 (GPR43) in gut immune cells and eliciting anti-inflammatory and tumor immunoregulatory effects (Guo et al., 2021). A recent study found that polysaccharides from *Polygonatum kingianum* reinforced quorum sensing signaling in *Ligilactobacillus faecis*, and regulated the expression of genes and proteins related to the synthesis and metabolism of SCFAs, including upregulating the *ldh* gene, *metE* gene, and adh2 protein and downregulating the *mvK* gene to impact the production of intestinal SCFAs (Yang et al., 2021). Most studies of the impact of herbal ingredients on the metabolic activity of LAB are focused on the SCFAs. Meanwhile, the effect of other immunoactive ingredients, such as EPS and bacteriocins, in herbal medicines remains to be explored.

## Immunoregulatory mechanism of lactic acid bacteria-fermented herbal medicines

Lactic acid bacteria-fermented herbal medicine primarily alters human immune function by activating innate immune responses, regulating adaptive immune responses, and maintaining intestinal immune homeostasis. Table 1 provides an overview of the immunomodulatory effects and molecular mechanisms of these treatments in various animal or cell models (Table 1).

### Activation of the innate immune system

**Figure 2:**
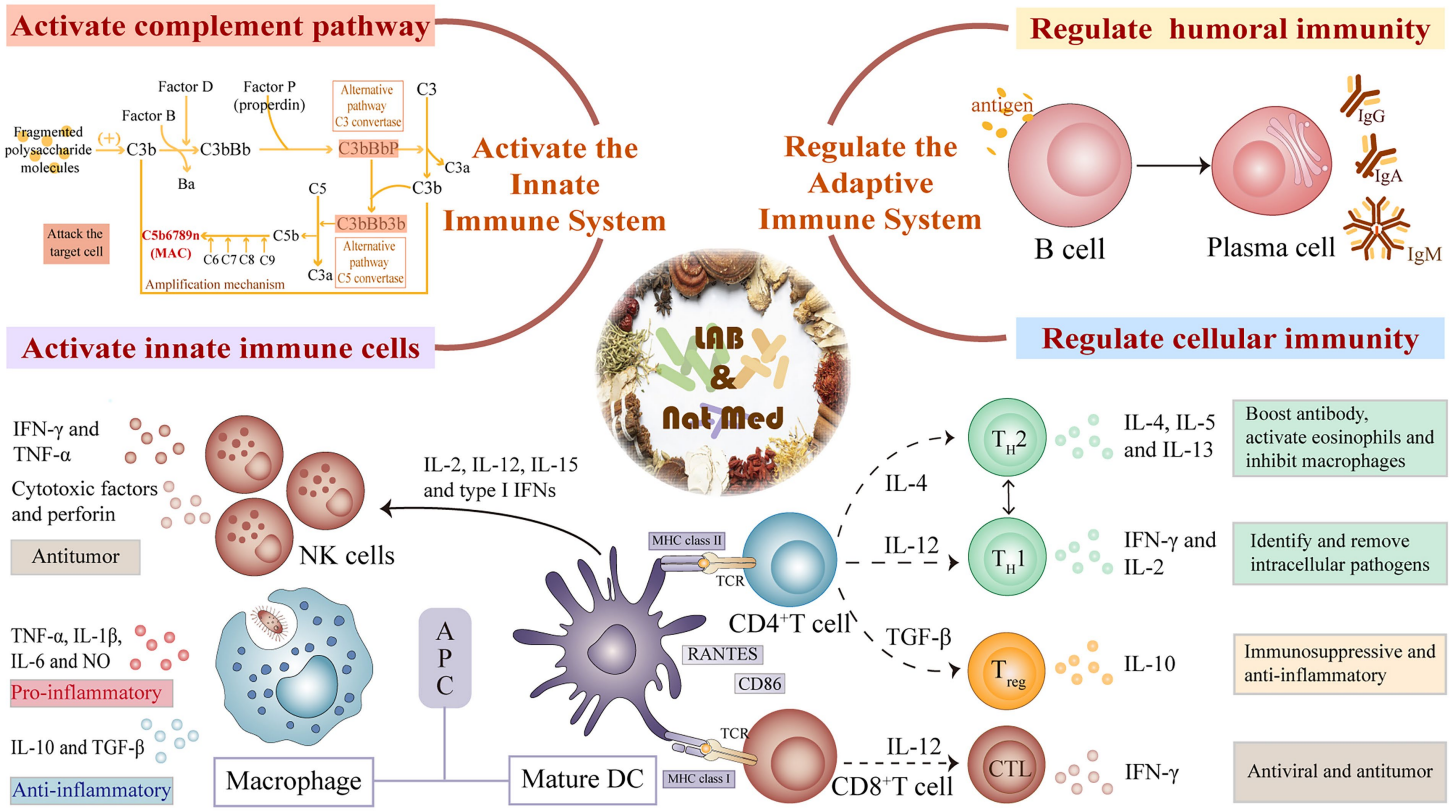
Mechanism of innate and adaptive immunity regulation of natural medicines fermented by lactic acid bacteria.

Components of the innate immune system, including its tissue barrier and innate immune cells and proteins, act as the first line of pathogen detection and clearance. LAB-mediated fermentation of natural medicines can improve immunity by activating innate immune cells, regulating immune-related cytokines, and activating the complement system (Figure 2).

#### Macrophages

Macrophages are a type of innate immune cells that play a critical role in maintaining homeostasis and resisting foreign pathogens (Lee et al., 2020). These cells act as immune effectors that directly eliminate foreign pathogens and tumor cells and are also integral antigen presenting cells (APCs), that activate immune responses by secreting pro- and anti-inflammatory cytokines and chemokines (Aminin and Wang, 2021). Macrophage Fcγ receptor (FcγR) promote phagocytosis by aggregating where these cells come into contact with exogenous antigens. LAB fermentation can promote phagocytosis by activating FcγR and TLR2 signaling (Kwon et al., 2018), and inducing macrophages to produce TNF-α and IL-6, cytokines involved in innate immune regulation (Wang and He, 2020). Kim et al. (2019) reported that after *Bifidobacterium animalis* subsp. *lactis*-induced fermentation of red ginseng, macrophage activity was significantly enhanced by mitogen-activated protein kinase (MAPK) and NF-κB signaling. Red ginseng fermentation also activated central immune cells such as splenic cells and bone marrow-derived macrophages (BMDM) by promoting the production of TNF-α and IL-6. NO secreted by macrophages also induces immune cell activation and promotes resistance to pathogens (Hu et al., 2021). Mo et al. (2021) observed that *Lacticaseibacillus paracasei* HY7017 cultivated in 3% RGE-supplemented medium dramatically boosted NO production. In addition, the mRNA level of iNOS, a key enzyme in NO synthesis, and COX-2 were higher after treatment with HY7017-RGEs. This process had no effect on the secretion of the anti-inflammatory cytokine IL-10, indicating that fermented red ginseng enhanced the immune response without creating excessive inflammation (Mo et al., 2021).

#### Dendritic cells

Dendritic cells (DCs) are a type of professional APCs that function as immune sentinels and help to initiate immune responses (Zanna et al., 2021). Immature DCs possess a strong migration ability, and mature DCs can effectively activate naive T cells, involved in the initiation, modulation and maintenance of immune responses (Zhou and Ye, 2021). Mature DCs synthesize high levels of NO, IL12, and the chemokine, RANTES, and express co-stimulatory molecules, such as CD86 and major histocompatibility complex (MHC), to induce full T cell activation (Zhu et al., 2019). Several studies have shown that LAB-fermented herbal drugs regulate immunity by promoting DC maturation, likely through the activation of MAPK-JNK, p38 MAPK, and NF-κB signaling pathways (Zhang et al., 2009). In a mouse model, FAPS was shown to impact DC maturation-related signaling, thereby promoting their maturation (Bian, 2017). The findings revealed that different doses of FAPS (50, 100, and 200 μg·mL^−1^) induced bone marrow-derived DCs to produce higher levels of IL-12, TNF-α, IL-1β, and IL-6 and related gene expression than unexposed DCs. By activating DCs, FAPS was also shown to promote 6.8–19% higher T cell proliferation (Bian, 2017). Another study found that DCs treated with fermented herbal medicines could directly stimulate B cell proliferation and differentiation. Zhang et al. (2009) reported that fermented Noni exudate (FNE) promoted the proliferation of mature DC and mixed spleen cell-cultured B lymphocytes, indicating that FNE can induce bone marrow-derived DC maturation and stimulate DC-induced immune responses required to promote B cell differentiation and immunoglobulin class conversion.

#### Natural killer cells

Natural killer (NK) cells, effector lymphocytes of the innate immune system, suppress tumors and microbial infections by patrolling for aberrant cells that lack MHC class I or overexpress NK cell receptors-specific ligands (Lanier, 2005; Vivier et al., 2008). After recognizing these cells, activated NK cells release granzymes and perforin to mediate target cell killing, and secrete several cytokines such as IFN-γ, TNF-α, and IL-10 that aid immunomodulation (Kim et al., 2007). Recent studies on autoimmune, transplantation and viral infections have linked NK cells to the control of T cell-mediated responses (Zwirner et al., 2021). LAB and its fermented products are shown to enhance NK cells activity and cytotoxicity. One study found that heat-killed *Lactobacillus acidophilus* La205 can directly stimulate NK cell cytotoxicity by increasing granule exocytosis (Cheon et al., 2011). Kang et al. (2010) assessed the effect of fermented ginseng extract on NK cell activity by measuring its cytotoxic impact on mouse lymphoma cells (Yac-1). The extract was shown to enhance NK cells cytotoxicity and promote IFN-γ secretion in a dose-dependent manner (Kang et al., 2010).

#### The complement system

The complement system involves a cascade of enzymatic reactions composed of ~40 serum proteins. There are three complement activation pathways, classical, alternative, and lectin (Garred et al., 2021). The alternative pathway, also known as the bypass pathway, directly activates C3 by microorganisms or foreign bodies and is not dependent on antibodies. The complement components, factors B, D, and P, help to induce a cascade of enzyme-catalyzed complement reaction pathways that lead to the production of C3 invertase and C5 invertase, the formation of membrane attack complex (MAC) and the destruction of target cells (Gupta and Tripathy, 2020). Fragmented polysaccharide molecules produced by LAB-fermentation are shown to activate complement through the alternative pathway and regulate immune responses (Watanabe et al., 2015; Mastuba et al., 2017; Chen et al., 2022).

### Regulation of the adaptive immune system

The innate immune system uses a limited number of germline-coded PRRs to recognize constant pathogen-associated molecular patterns (PAMPs). In contrast, the adaptive immune system depends on the production of multiple antigen receptors on T and B lymphocytes, and the subsequent activation and cloning of those cells with appropriate antigen-specific receptors (Schenten and Medzhitov, 2011). The active molecules in LAB-fermented natural drugs can modulate the immune response by altering humoral and cellular immune function (Figure 2).

#### Humoral immunity

Humoral immunity refers to adaptive immune defense mechanisms that are mediated by antibodies produced by B lymphocytes. Antigen stimulation transforms B cells into antibody-producing B cell lines and plasma cells that produce antibodies, namely immunoglobulin (Ig), that specifically bind to the antigen. IgG, an essential component of the humoral immune response, is the predominant Ig class present in human serum. IgG binding to Fc receptors on the surface of macrophages, neutrophils, and NK cells induces antibody-dependent cell-mediated cytotoxicity (ADCC) (Dixon et al., 2021). IgA is the most abundant immunoglobulin found in humans and exists in two forms, serotype and secretory (sIgA). IgA is primarily involved in maintaining mucosal homeostasis, primarily by interacting with IgA Fc receptor I (FcRI) (Mkaddem et al., 2014). IgM, a pentamer containing a joining chain polypeptide, is the first antibody produced after infection and is often regarded as the first line of immune defense (Hiramoto et al., 2018; Keyt et al., 2020). Red ginseng fermentation is shown to increase IgG and IgA levels in healthy human serum, potentially due to the presence of immunoreactive components such as polysaccharides and saponins (Kim et al., 2011). One study found that *Lp. Plantarum* fermentation of *Gynostemma pentaphyllum* oral liquid promoted the secretion of IgA, IgG, IgM and related cytokines into the serum of immunosuppressed mice and enhance their immunity (Du, 2021). These findings illustrate that LAB-fermented herbal medicines can enhance antigen- specific immune responses.

#### Cell-mediated immunity

Cell-mediated immunity is an immune response that is independent of antibodies but dependent on the ability of antigen-specific T cells to recognize and eradicate intracellular pathogens. Upon stimulation by their homologous antigen, immature T cells proliferate and differentiate into effector cells, and then undergo apoptosis or survive as precursors of long-lived memory cells. IL-2 is a potent T-cell growth factor that stimulates T-cell growth, proliferation, and subsequent differentiation and enhances their cytotoxic effects (Malek, 2003; Rochman et al., 2009). Activated T cells express TNFSF14, a member of the TNF superfamily that plays a critical role in the communication system that regulates T cell responsiveness (Wang and Fu, 2004). Kim et al. (2015) studied the effect of mixed fermentation broth on T cell activation by fermenting ginseng buds with *Lp. plantarum* and *Lacticaseibacillus casei*. This broth was shown to inhibit inflammation by preventing the expression of IL-1β, and enhancing the expression of IL-2 and TNFSF14 in T cells (Kim et al., 2015). There are two types of CD4^+^ T cells, T helper type 1 (Th1) and regulatory T cells (Treg). Th1 cells produce IFN-γ and IL-2 and induce cell-mediated immunity (Murakami et al., 2004), while Treg cells are essential for maintaining self-tolerance and immune cell homeostasis (Göschl et al., 2019). RG fermentation products can promote the differentiation of splenic lymphoid T cells into Th1 and Treg cells, reducing immunosuppression (Kim et al., 2018). The CD4^+^ / CD8^+^ ratio is a key indicator of immune function. *Lp. plantarum* NCU137-fermentation of Coix seed was found to significantly increase the splenic index, restore the CD4^+^/CD8^+^ ratio of splenic T lymphocyte subsets, down-regulate TNF- α and IL-6 production, and reduce inflammation, and the effect was better than that of unfermented group (Wang et al. 2022). Mixed probiotics (*Lactobacillus acidophilus* and *Bifidobacterium breve*)-mediated fermentation of *G. lucidum* fruiting bodies increased the proportion of CD4^+^ T and CD8^+^ T lymphocytes in mouse spleen and improved T cell transformation (Li et al., 2021).

### Maintenance of intestinal immune homeostasis

**Figure 3:**
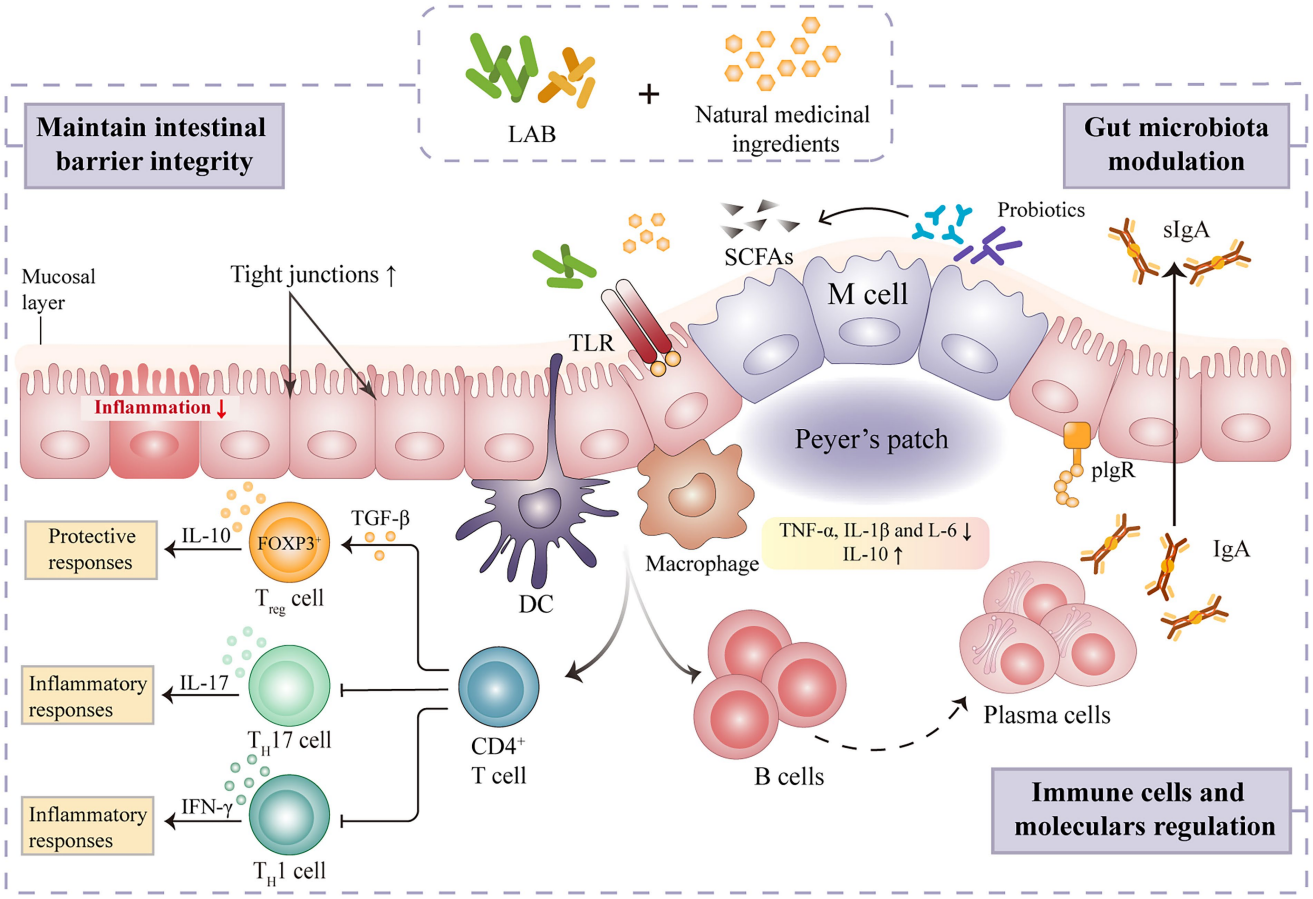
Mechanism of intestinal immune regulation of natural medicines fermented by lactic acid bacteria.

The intestinal tract is primarily composed of epithelial and immune cells, making it the largest immunological organ in the human body (Mahapatro et al., 2021). Gut immunity and homeostasis depend on a balanced gut microbiome. Different intestinal immune cells types are affected by microbial colonization, and gut immune cells can influence microbial population in both direct and indirect ways. LAB is thought to impact the intestinal barrier by modulating goblet cell-related genes such as mucin MUC2, trefoil factor 3, and resistin-like molecule β3 (Ren et al., 2018). Lactic acid fermentation is also shown to play a prominent role in regulating the regional structure of intestinal microorganisms, activating intestinal immune cells and factors, and alleviating inflammation (Figure 3). FAPS could restore the immune indexes and intestinal morphology of immunosuppressed mice (Liang, 2019). Indeed, sIgA, alpha chain and polymeric immunoglobulin receptor (pIgR) mRNA expression were all significantly up-regulated after dietary FAPS supplementation. FAPS can also promote the expression of tight junction (TJ) and adhesion junction (AJ) related proteins in the small intestine, maintaining the integrity of the intestinal epithelial barrier (Liang, 2019). A recent study showed that supplementation with fermented ginseng (390 mg·kg^−1^·day^−1^) significantly ameliorated lipopolysaccharide-induced inflammation in mice by activating TLR4/MAPK signaling and restoring the gut barrier (Fan et al., 2021). In addition, the immunomodulatory activity of LAB mediated fermentation of natural medicines correlates positively with the direct regulation of CD4^+^ T cell proliferation in intestinal Peyer’s patches (Li et al., 2021). Gong et al., (2020a) evaluated the effects of *Lp. plantarum* SHY001-fermented *Dendrobium candidum* (FDC) on gut microbial diversity and intestinal immune modulation in an oxazolone-induced model of intestinal inflammation in zebrafish. After FDC (0.15·g L^−1^) treatment, the abundance of beneficial bacteria, including *Lactobacillus*, *Faecalibacterium* and *Rummeliibacillus* increased, and the level of intestinal opportunistic pathogens, such as *Shewanella* and *Mycobacterium*, decreased (Gong et al., 2020a). FDC also significantly enhanced the production of intestinal SCFAs and the mucosal barrier-related proteins, MUC2, occludin, and ZO-1, and reduced the secretion of the inflammatory cytokines, IL-4, IL-10, and TNF-α (Gong et al., 2020a). These findings indicate that FDC can enhance intestinal health by modulating the gut microbiota and its metabolites in order to alleviate intestinal inflammation and regulate the host immune system.

## Conclusion

Herbal medicines have several immunoregulatory active ingredients, including polysaccharides, saponins, flavonoids and phenolic acids, that play a critical role in regulating immune function. Thus, the bioavailability of these components is essential for the immune-regulating efficacy of herbs. Recent studies have found that intestinal microorganisms greatly impact the metabolism and absorption of active medicinal substances (Wang S. et al., 2019; Gong et al., 2020b; Zhao et al., 2022). However, disordered immune functions are frequently accompanied by an imbalance in the intestinal flora that have a negative impact on drug absorption. LAB fermentation of herbal medicines *in vitro* is used as an effective strategy to assist drug absorption and metabolism. LAB played an important role in enhancing the immunoregulatory activity of fermented herbal medicine in three ways. First, LAB could produce immunomodulatory active substances, such as SCFAs, during the process of fermentation. Second, LAB-fermentation promotes the release of the original type immunoactive substances from herbal medicines. Third, LAB may induce the production of novel immunomodulatory compounds through the structural modification of particular components of these drugs.

Lactic acid bacteria are widely distributed in fermented foods, plants, animals, and humans but not all strains can be used in the fermentation of herbal medicine. In general, plant-derived strains are most suitable for use as starter cultures since they are more resistant to plant components such as polyphenol (Lee and Paik, 2017). Compared to food fermentation, the ideal industrial starter suitable for herbal medicine fermentation is still in the research stage. Most strains currently used in herbal fermentation research were derived from fermented food and intestinal flora and some medicinal plant-derived endophytic or exogenous bacteria that are more suitable for herbal fermentation have not been fully studied and developed. In fact, it is common for endophytic microorganisms to induce secondary metabolism in host medicinal plants. Studies indicate that medicinal plant microbiota can affect the yield of host plant alkaloids, steroids, terpenoids, and other important medicinal ingredients (Pang et al., 2021). These microorganisms may contain key genes or enzymes that synthesize important functional secondary metabolites. Thus, isolating and screening endophytic or exogenous LAB from medicinal plants will be essential for the efficient fermentation of herbs. It is worth noting that LAB activity is both species- and strain-specific. The metabolic activity of LAB is also affected by external factors such as fermentation substrate (type of herb) and culture conditions (time, temperature, pH, nutrient addition, etc.). These characteristics of LAB should be considered when screening culture starters for use in herbal medicine fermentation.

Current research on the immunomodulatory activity of LAB-fermented herbs is only focused on the role of herbal ingredients and does not account for the immunomodulatory properties of LAB, especially live bacteria. After oral administration of LAB-fermented herbs, LAB microbes can colonize the intestine and continue to secrete immunoregulatory active metabolites such as EPS, SCFAs, and bacteriocins, and have a strong and lasting impact on the immune system.

The mechanism by which LAB transforms or structurally modifies herbal ingredients has not been comprehensively and systematically studied. Research is instead primarily focused on hydrolysis, while demethylation and redox reactions are rarely reported. Metabolomics, transcriptomics, and other multi-omics techniques are gradually being applied to explore the biotransformation mechanisms of microbial fermented herbal medicines. However, the identification of novel metabolites with high activity and subsequent evidence of their role in immune modulation remain insufficient. In summary, the intricate interaction between LAB and herbal medicines offers new opportunities and challenges for the discovery of immunomodulatory components and the mechanism by which they modulate the immune response.

## Author contributions

HZ, LG, DY, and XD reviewed the literature and wrote the manuscript. HZ summarized and prepared the table and figures. All authors approved the submitted version.

## Conflict of interest

The authors declare that the research was conducted in the absence of any commercial or financial relationships that could be construed as a potential conflict of interest.

## Publisher’s note

All claims expressed in this article are solely those of the authors and do not necessarily represent those of their affiliated organizations, or those of the publisher, the editors and the reviewers. Any product that may be evaluated in this article, or claim that may be made by its manufacturer, is not guaranteed or endorsed by the publisher.
